# Gender Disparities in Health Biomarkers, Lifestyle Patterns, and Nutritional Status among Bank Staff: A Cross-Sectional Study

**DOI:** 10.3390/medicina60030413

**Published:** 2024-02-28

**Authors:** Markus Schauer, Martin Burtscher, Mohamad Motevalli, Derrick Tanous, Susanne Mair, Katharina Wirnitzer

**Affiliations:** 1 Department of Sport Science, University of Innsbruck, 6020 Innsbruck, Austria; 2 Department of Research and Development in Teacher Education, University College of Teacher Education Tyrol, 6010 Innsbruck, Austria; 3 Research Center Medical Humanities, University of Innsbruck, 6020 Innsbruck, Austria; 4 Department of Pediatric Oncology and Hematology, Charité—Universitätsmedizin Berlin, 10117 Berlin, Germany

**Keywords:** sex, nutrition, vitamin, mineral, homocysteine, coenzyme Q10, physical activity, inactivity, lifestyle, occupational health

## Abstract

*Background and Objectives*: Despite the importance of nutritional status and a healthy lifestyle in shaping overall well-being, little is known about examining gender-specific differences and trends in health, lifestyle, and nutritional status. The present study aimed to evaluate blood levels of micronutrients, homocysteine, and CoQ10, as well as physical activity (PA) levels and sedentary behavior, among a cohort of Austrian bank staff, with a particular focus on identifying gender differences as well as gender-specific nutritional deficiencies compared to the reference ranges. *Materials and Methods*: Following a cross-sectional study design, 123 Austrian bank staff (mean age: 43 years; 51% females) participated in this study. Blood samples were collected to evaluate participants’ micronutrient status and serum levels of homocysteine and CoQ10. Whole-blood values of macronutrients were compared to gender-specific reference ranges and categorized into three groups: below, within, or over the range. The WHO’s Global Physical Activity Questionnaire was used to assess PA levels and sedentary behaviors. *Results*: No significant difference between males and females was found for diet types, PA levels, sedentary time, homocysteine levels, or CoQ10 values (*p* > 0.05). A high PA level was reported by 64% of males and 58% of females. 71% of females and 56% of males were found to have a vitamin D deficiency. 63–98% of females and 72–97% of males showed normal blood levels for the remaining micronutrients, including potassium, calcium, magnesium, copper, iron, zinc, selenium, manganese, molybdenum, B_6_, B_9_, and B_12_. *Conclusions*: The findings highlight the necessity of implementing tailored strategies to foster healthy lifestyle behaviors, thereby enhancing the overall state of health, particularly in the context of occupational health.

## 1. Introduction

Health is the state of well-being that encompasses physical, mental, and social vitality, extending beyond the mere absence of illness [1]. Nowadays, non-communicable diseases (NCDs), including cardiovascular diseases, metabolic disorders, musculoskeletal dysfunctions, mental health conditions, and different types of cancer, have emerged as a significant global health burden, accounting for a vast proportion of morbidity and mortality worldwide [2]. A sedentary lifestyle, coupled with poor dietary habits, has significantly contributed to the rise in prevalence of NCDs, making it a critical public health concern [3]. Promoting preventive measures is essential to improving public health and reducing the burden of NCDs.

Occupational health is crucial for ensuring the well-being and safety of working populations, promoting their productivity, as well as fostering a positive work environment [4,5,6]. Today, the predominantly sedentary nature of most occupations contributes to a higher risk of NCDs and the increasing prevalence of obesity [6]. Data from the Austrian Health Survey 2019 indicate that nearly half of working-age individuals in Austria (44% of men and 46% of women) reported predominantly being physically inactive during their work hours [7]. Bank staff, for instance, often spend long hours seated at desks or in front of computer screens, engaging in mentally demanding tasks that could potentially foster unhealthy eating habits and exacerbate adverse health consequences [8]. Therefore, the occupational environment can profoundly influence health and well-being, even over a relatively short period of lifetime.

According to the Austrian Nutrition Report [9,10], diseases linked to nutrition (such as cardiovascular disorders, cancer, diabetes mellitus, and various chronic diseases) constitute a significant contributor to morbidity and mortality. While cancer is recognized as the primary cause of mortality for individuals under 70, cardiac disease is reported as the leading cause of death among those aged above 70 [9]. Assessing micronutrient status serves as a pivotal indicator for evaluating holistic health status and diagnosing chronic conditions [11,12,13]. Micronutrients, particularly vitamins and minerals, play crucial roles in various physiological processes, including but not limited to energy metabolism, immune function, antioxidant defense, DNA synthesis, neurotransmitter production, and hormone regulation, contributing significantly to the maintenance of biological homeostasis and the overall state of health [12,14]. Deficiencies or imbalances in essential micronutrients can lead to a wide range of health issues [12]. In the context of NCDs and obesity, specific micronutrient deficiencies have been associated with an elevated risk of health problems. For instance, inadequate levels of vitamin D have been associated with obesity and metabolic syndrome, which are risk factors for type 2 diabetes and cardiovascular diseases [15,16]. Additionally, deficiencies in minerals like magnesium, potassium, and calcium are linked to hypertension and cardiovascular issues [17]. Therefore, it is crucial to emphasize the importance of monitoring and addressing micronutrient status as an essential strategy for diagnosing nutritional deficiencies and reducing the risk of NCDs.

Besides micronutrients, additional biomarkers have been identified as co-indicators linked to health-related outcomes, including an increased risk of chronic diseases. Homocysteine, a non-proteinogenic amino acid derived from methionine biosynthesis, is recognized as a noteworthy biomarker of cardiovascular disease, potentially influencing atherogenesis [18,19]. Homocysteine metabolism is intricately regulated by a range of micronutrients, including vitamins B_6_, B_9_, and B_12_, and deficiencies in these micronutrients are associated with disruptions in the homocysteine metabolic pathway, leading to increased homocysteine levels and potential adverse health consequences [18,19]. Coenzyme Q10 (CoQ10) is an essential antioxidant and a critical component of the cellular respiration’s electron transport chain, playing a pivotal role in generating energy within cells [20]. It also acts as a potent scavenger of free radicals, safeguarding cells from oxidative damage [20,21]. The biosynthesis of CoQ10 relies on several micronutrients, including B-group vitamins, as well as specific minerals such as iron, zinc, and copper [21,22]. These facts highlight the significant interaction between micronutrients in regulating homocysteine levels and coenzyme Q10 biosynthesis, influencing the overall state of health. Regarding PA, while increased PA is known to be associated with lower blood homocysteine levels [23], there is a complexity in the relationship between PA and CoQ10 levels, which is influenced by a wide range of confounding factors [24], suggesting a need for additional investigations.

Gender differences in micronutrient status and health-related biomarkers have gained increasing attention due to their potential implications for specified health outcomes. Research has indicated that men and women may possess distinct dietary preferences and nutrient needs, resulting in disparities in micronutrient intake [25,26]. These differences, combined with their physiological variations, could impact their micronutrient status. For instance, women may necessitate higher iron and folate intakes due to menstruation and pregnancy [27], whereas men may require additional intake of specific micronutrients like zinc to maintain optimal testosterone levels and prostate health [28,29]. Moreover, research has demonstrated that sex hormones can impact the metabolism of homocysteine and CoQ10. Estrogen, for example, has been correlated with reduced homocysteine levels, potentially attributed to its role in amplifying the activity of enzymes engaged in homocysteine metabolism [30]. On the other hand, testosterone has been associated with elevated CoQ10 levels, highlighting a hormonal role in CoQ10 synthesis [31]. The influence of occupational stress may further alter hormonal and metabolic responses differently in men and women [32,33], ultimately leading to varied effects on their health and well-being. Regardless of the complex interplay between the above-mentioned factors, sex-specific differences in PA and sedentary behaviors may also contribute to divergent health outcomes between males and females [34,35,36], emphasizing the importance of tailored interventions to promote an active lifestyle. Therefore, understanding gender differences in micronutrient and health/lifestyle status is essential for developing effective strategies aimed at enhancing overall health and well-being.

Despite the increasing awareness of the pivotal roles that nutritional status and an active lifestyle play in shaping health outcomes, there remains a lack of research that specifically investigates the gender-specific disparities and trends in micronutrient and health status within the context of occupational health. The present study aims to address the gaps in the current body of knowledge by examining gender differentials in micronutrient profiles, PA levels, sedentary behaviors, as well as homocysteine and CoQ10 levels among bank staff, a susceptible cohort in terms of health vulnerability. This study also aims to shed light on the distinct nutritional deficiencies of male and female bank staff compared to the associated reference ranges. The ultimate goal is to provide valuable insights for designing tailored nutritional strategies to optimize health outcomes for populations working in sedentary workplaces.

## 2. Materials and Methods

### 2.1. Study Design and Participants

The present study followed a cross-sectional design. Austrian bank staff from the federal state of Tyrol, aged between 18 and 65, were voluntarily recruited to participate in the study. Only adult individuals employed by a Tyrolean bank were qualified to participate in the present study. The research protocol received ethical approval from the independent Ethical Board of the Medical University of Innsbruck (Ethikkommission Nr: 1136/2022), adhering to all international scientific and ethical guidelines, including the guidelines for Good Clinical Practice and the Helsinki Declaration. Data collection occurred during the routine, voluntary company health care program for Tyrolean bank employees and was conducted at the Biogena Diagnostics Point Tyrol. Participants were informed about the purpose, procedures, and potential risks and benefits of the research, and informed consent was obtained before including them in the study. The invitations highlighted the voluntary nature of participation, emphasizing that involvement in the study was entirely optional. Participants were explicitly informed of their right to withdraw from the study at any point without providing reasons. There was no remuneration for their participation in the study. An initial number of 280 participants were enrolled to participate; however, the final analysis included 123 participants who completed all the required measures, including blood tests and questionnaires. One participant, diagnosed with stomach cancer (a condition that may significantly affect nutritional status) was excluded from the final sample. Confidentiality of personal data was ensured throughout the study, and measures were put in place to anonymize and securely handle all collected information. Stringent ethical considerations were taken into account to safeguard the rights, privacy, and well-being of the study participants.

### 2.2. Measures

Two primary measures were utilized in this study. Firstly, blood samples were collected by a medical specialist at the Biogena Diagnostic Point Innsbruck to assess participants’ micronutrient status (including mineral and vitamin levels) as well as their serum levels of homocysteine and CoQ10. The blood analysis was conducted by GanzImmun, following standardized laboratory assessment protocols [37]. Secondly, a multi-module questionnaire was administered to 1. collect general data, including sociodemographic and anthropometric factors, work-related data, history of diseases, and dietary choices; 2. assess participants’ physical activity (PA) levels and sedentary behaviors. Participants filled out the printed version of the questionnaire within 20 min at the laboratory.

Body mass index (BMI; kg/m^2^) was calculated using self-reported body weight and height. Based on the World Health Organization BMI classifications for adults [38], participants were categorized into four BMI-based subgroups: underweight (<18.5 kg/m^2^), normal weight (18.5–25 kg/m^2^), overweight (25–30 kg/m^2^), and obese (>30 kg/m^2^). The questionnaire also inquired about participants’ diet types over the past four weeks using a direct question, supplemented by detailed explanations for each type (vegan, vegetarian, and omnivorous). Based on the self-reported diet types, participants were grouped into two categories: 1. omnivorous/mixed diet (no specific dietary restrictions), and 2. vegetarian (no meat and fish) or vegan diet (excluding all foods and ingredients derived from animal sources) [39,40].

### 2.3. Blood Analysis

The blood sampling procedure followed standard laboratory techniques to ensure the integrity of the samples. After a 12 h overnight fast, venous blood samples were drawn between 8:00 and 11:00 and collected with anticoagulant tubes. Each blood sample was bifurcated into two samples, with one of them designated for serum extraction. Following centrifugation (3 min, RT, at 3000 rpm), the serum was separated from the red blood cells, transferred to cryovials, and sent to the laboratory (GanzImmun) for further analysis within two hours.

Whole-blood samples were used to measure the levels of essential micronutrients, including potassium, calcium, magnesium, copper, iron, zinc, selenium, manganese, molybdenum, vitamin B_6_, vitamin B_9_ (folate), vitamin B_12_, and vitamin D, based on standardized laboratory assessment procedures [37]. Since numerous micronutrients are primarily distributed within blood cells, a comprehensive whole-blood analysis yields more significant insights compared to blood serum measurements [37,41,42]. It should also be considered that the vital biochemical functions of minerals and trace elements mostly occur at the cellular level [43], suggesting that serum values may not necessarily indicate sufficient cellular levels. Accordingly, it seems that by evaluating blood cells, primarily derived from the highly metabolically active bone marrow, a more precise strategy is employed to measure the metabolic status of these micronutrients. Therefore, a comprehensive assessment of blood cells was conducted to ensure obtaining a more accurate representation of micronutrient status.

Blood values of macronutrients were classified into three distinct categories: falling either below, within, or above the reference range. These intervals were established based on meticulously defined gender-specific reference ranges tailored for German-speaking populations [37]. This approach offers a thorough framework for comprehending and examining the wide range of macronutrient levels, and aids in the careful interpretation of micronutrient status.

The serum homocysteine concentration was measured using chemiluminescence immunoassay (CLIA) kits with a reagent detection range of 1–50 µmol/L. The measurement was carried out using the Centaur XPT analyzer by a senior laboratory technician. The intra-assay and inter-assay coefficients of variation for homocysteine samples were 3.9% and 5.8%, respectively. Serum CoQ10 levels were quantified using high-performance liquid chromatography (HPLC) with ultraviolet detection, ensuring accuracy and sensitivity in detecting these biomarkers. The CoQ10 values obtained from the participants’ blood samples were adjusted based on their cholesterol levels. Coenzyme Q10 is known to have a close relationship with cholesterol metabolism, as it is involved in the electron transport chain and plays a role in cholesterol synthesis [44]. Adjusting CoQ10 levels for cholesterol helps to account for individual variations in cholesterol metabolism, which may impact the circulating levels of CoQ10 [44]. To perform this adjustment, the CoQ10 values were divided by the corresponding cholesterol levels, resulting in a CoQ10/cholesterol ratio. Participants were classified into three groups based on their homocysteine levels: under 10 µmol/L, between 10 and 15 µmol/L, and above 15 µmol/L [45].

### 2.4. PA Questionnaire

The Global Physical Activity Questionnaire (GPAQ) was used to assess the participants’ PA levels and sedentary time. The GPAQ is a validated and widely used instrument developed by the World Health Organization (WHO) to collect comprehensive data on PA behavior in adult populations [46]. Participants were asked to complete the GPAQ questionnaire, providing information about their PA over a normal week. The questionnaire consists of four domains: work-related activities, transport-related activities, recreational activities, and sitting habits. For the analysis of PA levels, the GPAQ allowed participants to report the duration and frequency of activities classified as vigorous-intensity, moderate-intensity, and walking. The GPAQ utilizes a designated formula to determine participants’ PA levels. This process involves multiplying the reported durations of activities by their corresponding frequencies. Following the calculation of the total time spent at each intensity level, participants are categorized into one of three groups: those with low PA, indicating reported PA below the WHO’s recommended levels; those with moderate PA, meeting the guidelines for weekly PA; and those with high PA, surpassing the recommended levels, signifying a substantial amount of PA in their weekly routines [46,47]. The questionnaire also enabled participants to report their sedentary time, measured in hours per week, which included the time spent sitting or reclining during a typical day, excluding sleep.

### 2.5. Statistical Analysis

The statistical software R version 4.1.1 (R Foundation for Statistical Computing, Vienna, Austria) was utilized to conduct all statistical analyses. Exploratory analysis involved descriptive statistics, including mean values and standard deviation (SD) or median, range, and interquartile range (IQR). Normality of the numerical variables was assessed using the Kolmogorov–Smirnov test, ensuring a methodologically rigorous approach for subsequent analyses. Pearson chi-square tests (χ^2^) were employed for nominal scale data to examine the association of gender with BMI levels, diet type, PA levels, and homocysteine levels. For ordinal and metric scale variables, Kruskal–Wallis tests were approximated using t or F distributions, ordinary least squares, and standard errors (SE), with R^2^ to assess the association of gender with age, body weight, height, BMI, sedentary time, homocysteine, CoQ10, and adjusted CoQ10. Micronutrient values were categorized into three groups: below, within, and over the standard reference range, separately for females and males [35]. Box plots incorporating 95% confidence intervals were designed to illustrate potential gender differences in blood variables. Additionally, Likert plots were generated to depict the extent of variation in micronutrient values based on the reference ranges. The statistical level of significance was set at *p* ≤ 0.05.

## 3. Results

**Figure 1:**
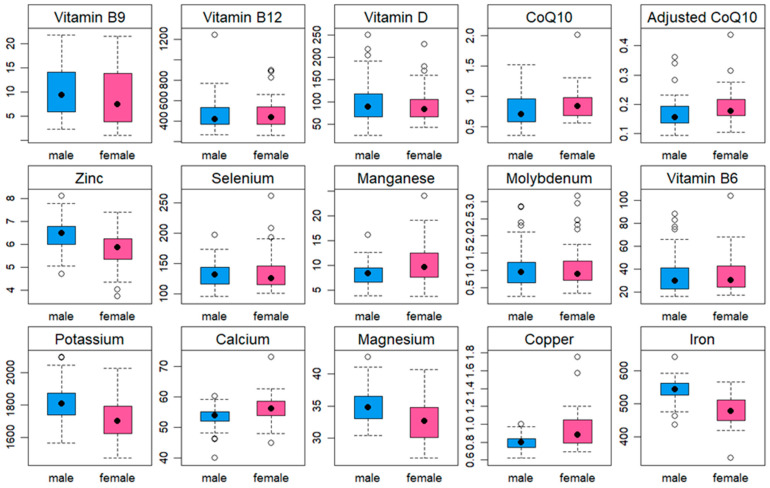
Box plots displaying the differences between females and males in blood variables.

The final statistical analysis was conducted on a sample of 123 adults (62 females and 61 males) with a median age of 43 years. Among the participants, 6% were classified as underweight, while the prevalence of overweight/obesity was 33%. The majority of participants (93%) reported following a mixed diet, while 7% identified as vegetarian or vegan. In terms of PA levels, 61% of participants had a high PA level, while 21% and 18% had moderate and low PA levels, respectively. The average body weight for the total participants was 71 ± 14 kg, with male participants having an average weight of 78 ± 10 kg and female participants averaging 64 ± 14 kg. In terms of height, the average for the total participants was 172 ± 8 cm, with males having an average height of 178 ± 6 cm and females averaging 167 ± 7 cm. A significant difference between males and females was found in BMI (*p* < 0.001), with males having a higher BMI than females (24.4 vs. 22.1 kg/m^2^, respectively). Analysis of BMI categories showed a significant difference between males and females (*p* < 0.001); the prevalence of being underweight was higher among females (11% vs. 0%), while the prevalence of overweight/obesity was greater among males (45% vs. 23%). A slightly significant gender difference was found in adjusted CoQ10 (*p* = 0.019), with females having higher adjusted CoQ10 values than males (0.19 vs. 0.17 µmol/mmol Chol). No significant difference between males and females was found for diet type, PA levels, sedentary time, homocysteine values, homocysteine levels, or CoQ10 values (*p* > 0.05). Table 1 shows a summary of participant characteristics and presents the gender-based statistical comparison of the study variables. Figure 1 illustrates the variations between females and males for each blood variable.

In comparison with reference norms, 68–85% of females exhibited normal blood mineral status. Specifically, 85% of females were within the normal range for zinc (the highest percentage), while 68% of them fell within the normal range for copper (the lowest percentage). Regarding blood vitamin status, 98% of females were within the normal range for vitamin B_12_, while only 19% fell within the normal range for vitamin D, with 71% of females being below the standard range of vitamin D. Table 2 shows the comparison of the micronutrient status of females with reference values.

**Figure 2:**
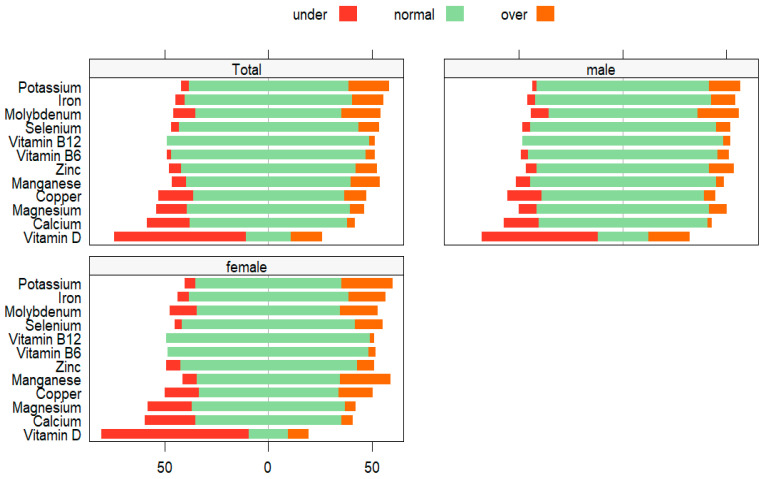
Likert plots displaying the extent of variation of micronutrient values from the gender-specific reference norms for German-speaking populations [35].

The comparison with reference norms revealed that 72–90% of males exhibited normal blood mineral status. Particularly, while 90% of males had normal levels of selenium and manganese, 72% fell within the normal range for molybdenum. Regarding blood vitamin status, the percentage of males within the normal range varied widely, ranging from 25% (for vitamin D) to 97% (for vitamin B_12_). Concerning vitamin D, 56% of males were lower than the standard range. Table 3 shows the micronutrient status of males based on the reference values. Figure 2 displays the extent of variation in micronutrient values from the reference norms.

## 4. Discussion

The present study aimed to assess gender differences in micronutrient status, PA levels, sedentary behavior, homocysteine, and CoQ10 levels among Austrian bank staff (*n* = 123), with a specific focus on identifying gender-specific nutritional deficiencies compared to local adult reference ranges. In general, it was found that: (i) male participants had a significantly higher BMI than females (24.4 vs. 22.1 kg/m^2^), but no significant gender differences were found for diet type, PA levels, sedentary time, homocysteine values, homocysteine levels, or CoQ10 values; (ii) despite being statistically insignificant, a high PA level was reported by 64% of males and 58% of females, while 16% of males and 26% of females had a moderate PA level, and 20% of males and 16% of females reported a low PA level; (iii) regarding micronutrient levels, except for vitamin D (where only 19% of females and 25% of males were within the normal range), 63–98% of females and 72–97% of males showed normal blood levels for the remaining micronutrients (including potassium, calcium, magnesium, copper, iron, zinc, selenium, manganese, molybdenum, vitamin B_6_, vitamin B_9_, and vitamin B_12_); (iv) a substantial deficiency in blood vitamin D levels was observed, with 71% of females and 56% of males falling below the standard range.

### 4.1. Anthropometry

Sociodemographic results from the present study showed a gender difference in BMI, with male participants exhibiting a higher BMI average than females, despite both groups being within the normal range. This finding aligns with national [48] and international [49] reference reports, underlining a consistent trend of higher BMI values among male adults compared to females. Consistently, a twofold higher prevalence of overweight/obesity was observed among males compared to their female counterparts (45% vs. 23%, respectively). This finding is partially in line with other national and international reports (including the Global Nutrition Report–Austria), indicating a higher prevalence of overweight/obesity among male adults compared to females [48,49,50,51]. Similar results were also reported from a series of Austrian studies conducted on school pupils and teachers [52,53] and university students and staff [54,55], as well as two Brazilian investigations on bank employees [56,57]. However, a study conducted on Ghanaian bank employees shows that, although highly prevalent in general (55.6%), there was no significant gender difference in the prevalence of overweight/obesity [58]. This can be explained by a complex interplay of genetic, socio-cultural, and behavioral factors that influence body composition and adiposity differently across populations [59,60].

### 4.2. PA Level and Sedentary Time

The present study revealed that 61% of bankers exhibited a high level of PA, which is greater than the estimated prevalence of Austrian adults meeting sufficient PA levels (approximately 25%) reported by the WHO [61]. This remarkable contrast may be attributed to various factors, such as corporate wellness initiatives, access to recreational spaces in their work and/or residential areas, the demographic characteristics of the participants, as well as the potential bias regarding self-reported data. The findings, however, contrast with those from a large-scale study on German adults conducted by the Robert Koch Institute, where only 20% of 7671 German participants reported achieving the WHO weekly PA recommendations [62]. Nevertheless, results from a Brazilian study indicate that approximately half of bank employees (52.2%) were physically active, with no significant difference between males and females [63]. These contradictory reports may also be associated with differences in PA assessment methods and the potential influence of sociocultural and environmental factors that may contribute to differential PA engagement.

Over the past decade, an increasing body of literature has emphasized the substantial morbidity risk associated with sedentary behavior, with some studies even equating the health risks of sedentary behavior to smoking [64,65,66]. The present study’s findings concerning sedentary time (42.5 h/week, equivalent to 364 min/day) were lower compared to results from a similar investigation on German adults (552 min/day) [67]. Interestingly, the German study also found that sedentary time was higher in males than in females [67], whereas in contrast, the present study observed no gender difference in sedentary time. Two separate studies examining bank employees in Nigeria and Ghana reported a prevalence of physical inactivity of 60% and 82%, respectively [68,69]. In this regard, a general trend of higher physical inactivity prevalence in females compared to males has also been reported [69,70,71]. An investigation on barriers to adopting healthy behaviors highlighted inadequate time for exercise as a major reason for the recent high levels of physical inactivity [72]. Notably, a recent meta-analysis reported that high amounts of sedentary behavior, assessed as sitting time, increased the risk of all-cause mortality, and this effect seemed to be mitigated through regular PA [73]. In general, gender-sensitive health promotion initiatives, taking into account the distinct PA and sedentary behavior patterns of males and females, are a crucial consideration that has been emphasized by the literature [74,75,76].

### 4.3. Homocysteine and CoQ10

In this study, we conducted an assessment of health biomarkers, specifically measuring blood levels of homocysteine and CoQ10. The goal was to gain insights into participant health status and emphasize the significance of gender-specific considerations within the context of occupational health. Hyperhomocysteinemia is characterized by homocysteine levels above 15 μmol/L and has been associated with several diseases, including an increased risk of cardiovascular problems [45,77,78]. In the present study, while there was no difference between males and females, 9% of participants were classified as having hyperhomocysteinemia, and 39% of participants were on the borderline of this risk (with blood homocysteine levels between 10 and 15 μmol/L). According to comparable data, hyperhomocysteinemia (Hcys > 15 micromol/L) was observed in 7% of German adults, and interestingly, it was found that males exhibited significantly higher homocysteine levels compared to females [79]. Nevertheless, the absence of a gender difference in homocysteine levels in the present study contradicts the available data, which typically report a higher prevalence of hyperhomocysteinemia and higher mean homocysteine concentrations in men compared to women [80,81,82]. Additionally, findings from a study conducted on Austrian youth highlight that sex is one of the most significant predictors of homocysteine levels [83]. Although no specific study has examined homocysteine levels among participants, an investigation conducted on Brazilian bank employees revealed a significant gender disparity in the prevalence of metabolic syndrome, which is considered a key health indicator, with a higher proportion of males (27.8%) being affected by metabolic syndrome compared to females (17.1%) [84].

In the present study, a slight but significant gender difference in cholesterol-adjusted CoQ10 values was observed, with female participants exhibiting higher adjusted CoQ10 values compared to males. This difference in cholesterol-adjusted CoQ10 values may be influenced by various factors, including hormonal variations, dietary habits, and metabolic processes specific to each gender. While the clinical implications of this discrepancy are required for further explanation, such differences underline the importance of considering gender as a potential modifier in the assessment of health biomarkers. Irrespective of gender, upon examining the levels of homocysteine, CoQ10, and adjusted CoQ10 across different PA levels (low, moderate, and high), we found no significant association between PA level and the aforementioned health markers. These findings collectively emphasize the importance of gender-specific considerations in occupational health assessments and lay the groundwork for further investigations into the associated indicators.

### 4.4. Micronutrients

The present study also investigated the micronutrient status of participants to gain insights into the blood mineral and vitamin levels of participants, with a focus on gender-specific patterns. When compared to reference norms [37], both male and female bank staff displayed variations in their blood mineral and vitamin statuses. Notably, with the exception of vitamin D (where only a small proportion of males and females were in the normal range), the blood levels of other micronutrients (including potassium, calcium, magnesium, copper, iron, zinc, selenium, manganese, molybdenum, vitamin B_6_, vitamin B_9_, and vitamin B_12_) were found to be within the normal range for a considerable portion of the studied population: 63–98% for females and 72–97% for males. As indicated by the German Society for Nutrition Report [85] and the Austrian Nutrition Reports of 2012 and 2017 [9,10], it is worth noting that both genders face challenges in adequately absorbing a range of minerals and vitamins (such as vitamin D, vitamin A, vitamin B_12_ and B_9_, as well as minerals like magnesium, potassium, calcium, and iodine) from regular dietary intake.

In the present study, it was observed that 71% of females and 56% of males fell below the standard range for vitamin D levels. This finding highlights the widespread problem of suboptimal vitamin D status within the Austrian population, a trend that aligns with findings from a similar study involving Austrian adults [86]. In addition, numerous studies worldwide have consistently documented low vitamin D levels [87,88,89], indicating the universal nature of this issue, which emphasize the necessity for focused strategies to tackle vitamin D deficiency. In line with the present findings, results from a systematic review involving a pooled analysis of 7.9 million participants showed that the prevalence of vitamin D deficiency was generally higher in females than in males [87]. However, the validity of the cutoff points used to define vitamin D deficiency across diverse populations is subject to scrutiny [86,90]. Additionally, it has been reported that seasonal changes strongly influence vitamin D status, with improved levels during the summer and early autumn months, which highlights the significance of endogenous vitamin D synthesis [86]. Furthermore, while the small representation of vegans/vegetarians in the present study hinders a thorough analysis of their potential impacts, it has been documented that dietary preferences can serve as indicators for vitamin D levels [91]. Research indicates that vegans and vegetarians might be more susceptible to vitamin D deficiency compared to nonvegetarians, given the scarcity of natural vitamin D in most plant foods [92]. The present findings collectively underline the importance of examining nutrient status and the need for tailored interventions to address specific nutrient deficiencies, particularly vitamin D, as the most critical concern among both genders. However, the study’s cross-sectional nature limits the ability to establish causal relationships, and further research is needed to elucidate the underlying factors contributing to these nutrient status discrepancies.

### 4.5. Implications of the Study

The present study contributes to the limited body of knowledge by addressing a specific gap in understanding the health dynamics of adult populations with a sedentary job nature. In general, the findings of this study carry significant implications for both public health and workplace interventions within the context of occupational health. The identification of widespread vitamin D deficiencies, particularly among both male and female participants, highlights the urgent need for public health initiatives aimed at enhancing vitamin D status in the general population. In this regard, education campaigns and policy measures to promote sunlight exposure and dietary supplementation could play a pivotal role in mitigating this concern. Additionally, the assessment of PA levels highlights the importance of promoting an active lifestyle among bank staff. Workplace interventions, such as structured exercise programs and ergonomic modifications, could encourage increased PA and help mitigate sedentary behavior. The gender-specific findings regarding micronutrient statuses call for collaborative efforts between employers and health professionals to facilitate the provision of diverse dietary options that address individual needs and promote optimal health. Health promotion programs should consider the unique needs of male and female bank staff, targeting factors such as micronutrient intake, PA, and cardiovascular health. Moreover, regular health screenings and educational workshops within the workplace could empower bank staff with personalized health insights and information. These initiatives could enhance greater awareness about nutrient deficiencies, cardiovascular risk factors, and lifestyle choices, fostering a culture of proactive health management.

### 4.6. Limitations, Strengths, and Recommendations for Future Research

The present study has notable limitations that need to be considered. The study’s cross-sectional design limits its ability to establish causal relationships between the study variables and health outcomes. A limitation regarding the BMI calculation was its reliance on self-reported body weight and height, which can introduce inaccuracies due to potential misreporting. This may lead to misclassification of weight status, necessitating caution in interpreting the associated data. Additionally, the utilization of a survey-based approach and dependency on self-reported data regarding PA (despite the validation of the PA questionnaire) highlight the possibility of recall and reporting biases, leading to underreporting or overreporting of certain behaviors in line with participant desires and influencing the accuracy of the obtained data. The study exclusively focused on bank staff, which may potentially restrict the generalizability of the findings to other occupational sectors, while a more diverse sample encompassing various occupations could offer a broader perspective on the observed findings. Moreover, certain influential variables, such as socioeconomic status, specific job roles, and comorbidities, which could contribute to confounding effects, were not considered in the analysis.

Despite these limitations, a strength of this study lies in its comprehensive assessment of multidimensional health parameters, leading to a holistic understanding of the health profile among the target group. Furthermore, the study places emphasis on gender-specific analyses, recognizing the potential for sex-specific considerations in health outcomes. This result underlines the importance of tailored interventions that address the specific needs of both male and female bank staff. Future research could employ longitudinal study designs to unravel the temporal relationships between occupational factors, health parameters, and well-being outcomes. This approach would provide deeper insights into the impact of work-related variables on health trajectories over time. From a methodological viewpoint, future studies can benefit from incorporating tailored methods to assess dietary patterns, utilizing advanced body composition measurements beyond BMI, and integrating quantitative tools to assess PA patterns. These refinements to the methodology promise to enhance the reliability and depth of insights into the relationships between lifestyle factors and health outcomes. Extending the investigation to other occupational sectors would also allow for broader comparisons and a more comprehensive understanding of how health parameters vary across diverse work environments. Furthermore, evaluating the effectiveness of workplace interventions, whether aimed at improving PA, dietary habits, or cardiovascular health, would contribute to developing evidence-based strategies that can be implemented on a larger scale.

## 5. Conclusions

The present study reveals that a majority of both female and male bank staff exhibit optimal levels of blood micronutrients (as determined by whole-blood analysis) and physical activity (as assessed using the WHO-GPAQ), when compared to existing literature data concerning Austrian or German-speaking adults. Nonetheless, it is noteworthy that a substantial proportion of the target population (71% of females and 56% of males) demonstrate a deficiency in vitamin D, emphasizing the need for targeted interventions to address this widespread deficiency. The findings of this study hold implications for both public health and workplace interventions, highlighting the need for targeted strategies to improve the health and well-being of bankers. By addressing micronutrient deficiencies, promoting PA, and fostering a health-conscious work environment, future initiatives can contribute to healthier lifestyles and ultimately enhance the overall quality of life for individuals within the occupational sector.

## Figures and Tables

**Table 1:** Summary of participant characteristics and statistical comparison between males and females.

		Total (*n* = 123)	Females (*n* = 62)	Males (*n* = 61)	Statistics and *p*-Values (Gender Differences)
Age (years)		43 (20–65)	41 (20–65)	44 (23–65)	F_(1,121)_ = 0.78, *p* = 0.378
Body Weight (kg)		71 (43–114)	64 (43–114)	77 (53–114)	F_(1,121)_ = 55.53, *p* < 0.001
Height (cm)		173 (154–190)	167 (154–185)	178 (165–190)	F_(1,121)_ = 97.24, *p* < 0.001
BMI (kg/m^2^)		23.6 (16.9–40.4)	22.1 (16.9–40.4)	24.4 (19.0–36.0)	F_(1,121)_ = 14.79, *p* < 0.001
BMI Levels	<18.5	6%	11%	-	χ^2^_(3)_ = 18.57, *p* < 0.001
	18.5–25.0	61%	66%	56%	
	25.0–30.0	28%	15%	43%	
	>30.0	5%	8%	2%	
Diet Type	mixed	93%	90%	95%	χ^2^_(1)_ = 1.03, *p* = 0.311
	vegetarian/vegan	7%	10%	5%	
PA Levels	low	18%	16%	20%	χ^2^_(2)_ = 1.68, *p* = 0.432
	moderate	21%	26%	16%	
	high	61%	58%	64%	
Total MET		8598 (13,526)	7290 (14,606)	9927 (12,311)	F_(1,121)_ = 1.84, *p* = 0.177
Work MET		4889 (9949)	3644 (8897)	6155 (10,842)	F_(1,121)_ = 1.21, *p* = 0.273
Transport MET		313 (1866)	337 (2139)	290 (1557)	F_(1,121)_ = 0.12, *p* = 0.727
Recreation MET		3395 (4448)	3310 (4561)	3482 (4366)	F_(1,121)_ = 0.24, *p* = 0.622
Sedentary Time (h/week)		42.5 ± 23.3	40.7 ± 22.1	44.3 ± 24.5	F_(1,121)_ = 0.70, *p* = 0.404
Homocysteine (µmol/L)		10.6 ± 4.3	10.67 ± 5.36	10.47 ± 2.90	F_(1,121)_ = 0.73, *p* = 0.395
Homocysteine Levels	<10	52%	58%	46%	χ^2^_(2)_ = 3.89, *p* = 0.143
	10–15	39%	31%	48%	
	>15	9%	11%	7%	
CoQ10 (mg/L)		0.82 ± 0.28 *	0.87 ± 0.29	0.78 ± 0.28	F_(1,80)_ = 2.58, *p* = 0.112
Adjusted CoQ10 (µmol/mmol Chol)		0.18 ± 0.06 *	0.19 ± 0.06	0.17 ± 0.05	F_(1,80)_ = 5.75, *p* = 0.019

* Total number of participants: 82. BMI: body mass index; PA: physical activity; MET: metabolic equivalent of task; CoQ10: coenzyme Q10. Data are presented as percentage, median (with range), or mean ± standard deviation.

**Table 2:** Micronutrient status of female participants and comparison with reference values.

	Reference Range for Females *	Females (*n* = 62)			
		Value	Below the Range	Within the Range	Over the Range
Potassium (mg/L)	1484–1794	1687 ± 234	5%	71%	24%
Calcium (mg/L)	53.8–62.7	56.3 ± 4.1	24%	71%	5%
Magnesium (mg/L)	29.8–37.5	32.8 ± 3.0	21%	74%	5%
Copper (mg/L)	0.76–1.12	0.93 ± 0.20	16%	68%	16%
Iron (mg/L)	423–520	482 ± 43	5%	77%	18%
Zinc (mg/L)	4.88–6.67	5.80 ± 0.69	6%	85%	8%
Selenium (µg/L)	101–170	138 ± 42	3%	84%	13%
Manganese (µg/L)	5.91–12.7	10.33 ± 3.70	6%	69%	24%
Molybdenum (µg/L)	0.5–1.6	1.12 ± 0.83	13%	69%	18%
Vitamin B_6_ (µg/L)	16.4–80.4	38.2 ± 30.8	-	97%	3%
Vitamin B_9_ (ng/mL)	>5.38	8.88 ± 5.70	37%	63%	-
Vitamin B_12_ (pg/mL)	211–911	579 ± 877	-	98%	2%
Vitamin D (nmol/L)	100–150	89.9 ± 37.2	71%	19%	10%

* Values are based on gender-specific reference norms for German-speaking populations [35].

**Table 3:** Micronutrient status of male participants and comparison with reference values.

	Reference Range for Males *	Males (*n* = 61)			
		Value	Below the Range	Within the Range	Over the Range
Potassium (mg/L)	1568–1908	1811 ± 113	2%	84%	15%
Calcium (mg/L)	50.3–59.8	53.5 ± 3.4	16%	82%	2%
Magnesium (mg/L)	31.2–39.1	34.9 ± 2.8	8%	84%	8%
Copper (mg/L)	0.7–0.94	0.79 ± 0.09	16%	79%	5%
Iron (mg/L)	465–577	543 ± 34	3%	85%	11%
Zinc (mg/L)	5.36–7.29	6.43 ± 0.68	5%	84%	11%
Selenium (µg/L)	101–168	136 ± 35	3%	90%	7%
Manganese (µg/L)	5.39–11.2	8.29 ± 2.13	7%	90%	3%
Molybdenum (µg/L)	0.45–1.56	1.60 ± 4.23	8%	72%	20%
Vitamin B_6_ (µg/L)	16.4–80.4	36.5 ± 22.3	3%	92%	5%
Vitamin B_9_ (ng/mL)	>5.38	10.02 ± 4.92	15%	85%	-
Vitamin B_12_ (pg/mL)	211–911	491 ± 247	-	97%	3%
Vitamin D (nmol/L)	100–150	99.8 ± 49.9	56%	25%	20%

* Values are based on gender-specific reference norms for German-speaking populations [35].

## Data Availability

Individual-level data cannot be available due to privacy and ethical restrictions. The data that support the findings of this study are available upon reasonable request from the corresponding author.
